# Three types of *Leishmania mexicana* amastigotes: Proteome comparison by quantitative proteomic analysis

**DOI:** 10.3389/fcimb.2022.1022448

**Published:** 2022-11-09

**Authors:** Lenka Pacakova, Karel Harant, Petr Volf, Tereza Lestinova

**Affiliations:** ^1^ Department of Parasitology, Charles University, Prague, Czechia; ^2^ Biotechnology and Biomedicine Centre of the Academy of Sciences and Charles University (BIOCEV), Vestec u Prahy, Czechia; ^3^ Proteomics Core Facility, Faculty of Science, Charles University, Prague, Czechia

**Keywords:** proteome, *Leishmania (L) mexicana*, amastigote, axenic, macrophage, lesion, tandem mass tags labeling

## Abstract

*Leishmania* is the unicellular parasite transmitted by phlebotomine sand fly bite. It exists in two different forms; extracellular promastigotes, occurring in the gut of sand flies, and intracellular, round-shaped amastigotes residing mainly in vertebrate macrophages. As amastigotes originating from infected animals are often present in insufficient quality and quantity, two alternative types of amastigotes were introduced for laboratory experiments: axenic amastigotes and amastigotes from macrophages infected *in vitro*. Nevertheless, there is very little information about the degree of similarity/difference among these three types of amastigotes on proteomic level, whose comparison is crucial for assessing the suitability of using alternative types of amastigotes in experiments. In this study, *L. mexicana* amastigotes obtained from lesion of infected BALB/c mice were proteomically compared with alternatively cultivated amastigotes (axenic and macrophage-derived ones). Amastigotes of all three types were isolated, individually treated and analysed by LC-MS/MS proteomic analysis with quantification using TMT^10^-plex isobaric labeling. Significant differences were observed in the abundance of metabolic enzymes, virulence factors and proteins involved in translation and condensation of DNA. The most pronounced differences were observed between axenic amastigotes and lesion-derived amastigotes, macrophage-derived amastigotes were mostly intermediate between axenic and lesion-derived ones.

## Introduction

*Leishmania* spp. are protozoan parasites from the trypanosomatid family and causative agents of wide spectrum of diseases called leishmaniases. More than 1 billion people are living in areas of risk, with an estimated 0.7-1million new cases annually (Burza et al., 2018; Ruiz-Postigo and Grout, 2020).

The manifestation and severity of the diseases varies depending on the species and strain of *Leishmania* parasites as well as on the genetic background and the state of host immune system (Loeuillet et al., 2016). New World species *Leishmania mexicana*, used in this study, is typically associated with cutaneous leishmaniasis, the most common form of this disease, usually manifested by an ulcer that develops at the site of inoculation. While lesion is usually self-healing, it leaves life-long stigmatizing scar (Steverding, 2017; Maxfield and Crane, 2019).

The life cycle of *Leishmania* is digenetic; it alternates between a vertebrate host and an insect vector, a female phlebotomine sand fly of the genera *Phlebotomus* (Old World) or *Lutzomyia* (New World) (Maroli et al., 2013). After transition from the insect vector to the mammalian host, the extracellular elongated motile promastigotes transform into round, aflagelated, non-motile intracellular amastigotes that reside in phagolysosome of a mammalian macrophage. This differentiation is triggered by temperature, pH, availability of nutrients and results in changes in gene expression including virulence factors and metabolism (Coombs et al., 1982; Hart and Coombs, 1982; Nugent et al., 2004; Paape et al., 2008; McConville et al., 2015).

Transformation to amastigote form is associated with changes in carbon source utilization. Fatty acids, glucose and amino acids in particular are important sources of carbon for amastigotes (Hart and Coombs, 1982). Despite the necessity of these nutrients, their intake is reduced in amastigotes compared to promastigotes (Saunders et al., 2014). In amastigotes living naturally in sugar-poor conditions, the glycolysis is downregulated while gluconeogenesis is upregulated and remains active even when the sugar is available (Rodríguez-Contreras and Landfear, 2006). Catabolic pathways like β-oxidation of fatty acids, oxidative phosphorylation, tricarboxylic acid cycle (TCA) and amino acid oxidation are also upregulated in amastigotes (Mottram and Coombs, 1985; Nugent et al., 2004; Lahav et al., 2011). Inhibition of TCA or glutamine synthase leads to growth arrest in both axenic and macrophage-derived amastigotes (Saunders et al., 2014).

These inter-stage changes in metabolism are not exclusively due to the availability of different nutrients, but this effect is rather tied to various signals. Amastigotes cultivated axenically *in vitro* possessed the same pattern of changes as lesion-derived amastigotes despite the abundance of nutrients in medium (Saunders et al., 2014).

While the cultivation of promastigotes is relatively simple and easily performed in an appropriate medium, a study of amastigote form lags behind due to complicated isolation from the host tissue resulting in low numbers of parasite and massive host tissue contamination. Therefore, a method of axenic cultivation has been developed for some *Leishmania* species including *L. mexicana* used in present study (Bates et al., 1992; Pan et al., 1993; Debrabant et al, 2004).

Axenic amastigotes of *L. mexicana* are prepared by imitating the environment of phagolysosome by lowering pH and subsequent cultivation in higher temperatures (Bates et al., 1992; Bates and Tetley, 1993). Advantage of this method is that it reduces the use of laboratory animals and the contamination by host tissue and it provides high numbers of amastigotes. Axenic amastigotes are criticized because they are not equal to lesion-derived amastigotes most likely because the process *in vitro* is not exactly reflecting what happens in macrophages (Holzer et al., 2006; Pescher et al., 2011). For this reason, amastigotes isolated from *in vitro* infected macrophages have been used in some studies with experimentally infected sand flies (Sadlova et al., 2017; Pruzinova et al., 2018). This type of amastigote undergoes selective pressure of a macrophage, but still is not exposed to any other component of host immunity.

Studies comparing alternative sources of amastigotes with natural ones, isolated from infected hosts, are very limited, focused either only on its external features (morphology, ultrastructure) (Pan and Pan, 1986; Eperon and Mcmahon-Pratt, 1989; Bates et al., 1992; Pral et al., 1993; Ueda-Nakamura et al., 2007), or on their transcriptomes (Holzer et al., 2006; Fiebig et al., 2015). Nevertheless, regulation of protein expression in trypanosomatids is mediated largely post-transcriptionally (Ivens et al., 2005; Peacock et al., 2007; Lahav et al., 2011; De Pablos et al., 2019) and therefore, it is essential to compare proteomes rather than genomes and transcriptomes to understand complex processes such as cellular function or disease outcome.

We applied quantitative proteomics with isobaric labeling to compare the protein levels from axenically cultivated amastigotes, macrophage-derived and lesion-derived amastigotes and revealed differences in virulence factors and metabolic pathways like glycolysis, fatty acid or amino acid metabolism and other cellular processes like vesicular trafficking.

## Materials and methods

### Ethics statement

BALB/c mice were maintained and handled in the animal facility of Charles University in Prague in accordance with institutional guidelines and Czech legislation (Act No. 246/1992 and 359/2012 coll. on Protection of Animals against Cruelty in present statutes at large), which complies with all relevant European Union and international guidelines for experimental animals.

The animal study was reviewed and approved by The Committee on Ethics of Laboratory Experiments, Faculty of Science, Charles University, Czech Republic.

Investigators are certificated for experimentation with animals by the Ministry of Agriculture of the Czech Republic (certificate no. CZ 03744). All efforts were made to minimize the number and suffering of experimental animals during the study.

### Leishmania cell culture and amastigotes

*L. mexicana* (MNYC/BZ/62/M379) promastigotes were cultured in RPMI 1640 HEPES (Sigma-Aldrich) supplemented with 10% fetal bovine serum (FBS) (Sigma-Aldrich), 0.1% amikin (Sigma-Aldrich), 1% BME vitamins (Sigma-Aldrich) and 0.5% sterile human urine. For mice and macrophage infections low passage of parasites was used (maximally P4) due to its decreasing virulence caused by long-term passaging (Ali et al., 2013).

Macrophage-derived amastigotes were obtained as described previously (Pruzinova et al., 2018). Macrophages were differentiated from precursor cells of mouse bone marrow by adding macrophage colony stimulating factor (M-CSF) contained in L929 fibroblast supernatant. Cells were stimulated to transformation in 37°C and 5% CO_2_ for 7-10 days in RPMI 1640 HEPES supplemented with 20% M-CSF medium, 10% FBS, 50 mM mercaptoethanol, mixture of antibiotics and amino acids (L-glutamine 200 mM-peniciline 10 000 U-streptomycine 10 mg/ml), (Sigma-Aldrich).

Macrophages were infected with stationary phase of promastigotes in a ratio of 6 parasites per 1 macrophage. After 72h macrophages were disrupted by lysis buffer (M199 (Sigma-Aldrich) + 0,016% SDS for maximum of 7 minutes) and lysis was stopped by M199 medium supplemented with 20% FBS. Disruption was completed mechanically by rubbing a syringe plunger and amastigotes were released by repeated aspiration into a 1 ml insulin syringe and washed three times by centrifugation at 3010 x g for 10 min.

Axenic amastigotes were established from amastigotes obtained from *in vitro* infected macrophages. Macrophage-derived amastigotes were passaged into RPMI medium (described above) and cultivated at 23°C for reverse transformation to flagellated promastigotes. After 72h, promastigotes were kept in medium composed of Grace´s insect medium (Sigma-Aldrich) supplemented with 20% FBS (Sigma-Aldrich), amikin (0.1%) (Sigma-Aldrich), at pH 5.4 and temperature of 25°C for 6 days to induce metacyclogenesis (Bates and Tetley, 1993). Subsequently, metacyclic promastigotes were transferred into fresh „amastigote RPMI” medium and cultivated in 33°C (Bates, 1994). First passage of axenic amastigotes was collected after nine days and further subpassaged every 7-10 days.

Lesion-derived amastigotes were obtained from BALB/c mice infected with 1x10^7^ of promastigotes from a culture in stationary phase of growth and injected subcutaneously to the base of tail in 50 μl sterile saline.

Mice were sacrificed 20 weeks after infection. Lesion area was sterilized by 70% ethanol, excised under sterile conditions and homogenized using glass Potter homogenizer in saline. Larger pieces of tissue were removed by filtering through sterile monofile into polypropylene tube (Thermo Fisher Scientific) kept on ice. Homogenate was washed twice by centrifugation at 3010 x g for 10 min, 4°C and after second washing step, the pelett was resuspended in 2-4 ml of erythrocyte lysis buffer (155 mM NH_4_Cl, 10 mM KHCO, 3.1 mM EDTA, pH 7.4) for 2 min to eliminate red blood cells responsible for the host hemoglobin contamination (de Rezende et al, 2017). Lysis was stopped by repeated washing in sterile saline. Between individual washing steps, amastigotes were released by repeated aspiration to insulin syringe. Pellet was kept in -80°C until use.

**Figure 1:**
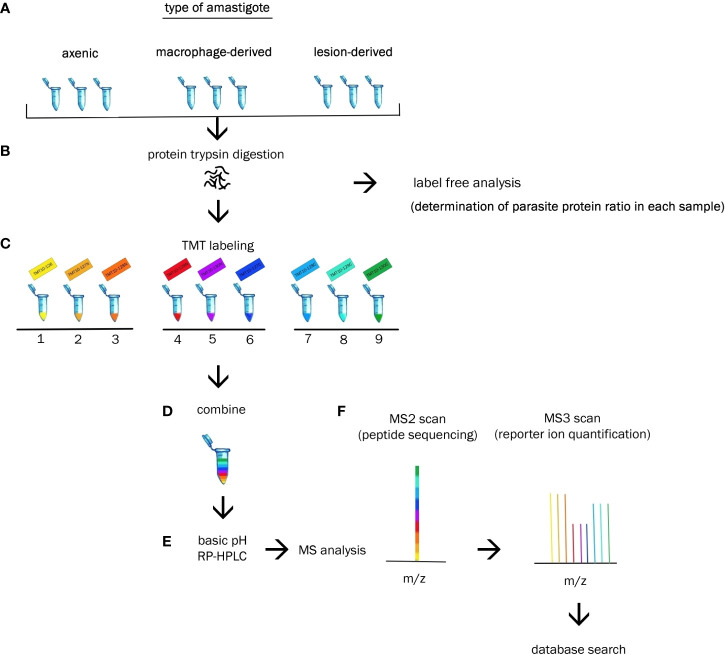
Experimental workflow. **(A)** Biologic triplicates of axenic, macrophage-derived and lesion-derived amastigotes were homogenized. **(B)** Extracted proteins from each sample were digested with trypsin and subjected to label free analysis to determine the percentage of *Leishmania* protein in each sample. **(C)** Each sample was labeled with a unique TMT10-plex reagent. **(D)** Resulting peptides were combined at equal ratios of parasite protein. **(E)** Peptides were fractionated *via* basic RP-HPLC separation. **(E)** Reversed-phase HPLC was performed prior to tandem MS analysis. **(F)** Identification and quantification of the proteins were performed using bioinformatics and statistical analysis.

Each amastigote type was analysed in triplicates during single measurement. Each sample of lesion amastigotes was isolated from different mouse. Axenic amastigotes were collected at three consecutive passages. Macrophage-derived amastigotes were obtained by using three consecutive passages of promastigote culture to initiate macrophage infection. Cultivation of each particular type of amastigote was performed under the same cultivation and isolation conditions. Each of the 9 samples was labeled with different isotopic variant of the label TMT 10-plex (**Figure 1**).

### Proteomic analysis

Cell pellets were lysed in 100 mM TEAB containing 2% SDC, 10 mM TCEP, and 40 mM chloroacetamide and boiled at 95°C for 5 min. Protein concentration was determined using BCA protein assay kit (Thermo) and 20 µg of protein per sample was used for MS sample preparation. Samples were digested with trypsine (trypsin/protein ratio 1:20) at 37°C overnight. After digestion, samples were acidified with TFA to 1% final concentration. SDC was removed by extraction to ethylacetate (Masuda et al, 2008) and peptides were desalted using in-house made stage tips packed with C18 discs (Empore) according to (Ishihama et al., 2006).

Samples were measured by LC/MS Orbitrap Fusion with protein label-free quantification with MaxQuant software (Cox et al., 2014). Ratios of the sum of intensities for mice proteins and *L. mexicana* proteins were determined for each sample to allow for subsequent normalization based on the amount of *L. mexicana* proteins. Tandem mass tag (TMT) reagents were added to each sample, according to the manufacturer’s protocol (Thermo Scientific Pierce), and after 60 min, the reaction was stopped by the addition of 0.5% hydroxylamine. Labeled samples were pooled in ratios determined from label-free quantification runs; as a result, the pooled sample contained equal amounts of *L. mexicana* proteins from each individual sample.

100 μg of peptides of pooled sample were injected on to C18 column (YMC 1.9 μm, C18, 300x0.3 mm) and separated with linear gradient from 0% A (of 20 mM ammonium formate, 2% acetonitrile pH 10) to 50% B (of 20 mM ammonium formate, 80% acetonitrile pH 10) in 60 minutes, flow 3μl/min. 64 Fractions were collected and pooled in to 8 fractions (Kulak et al., 2017). The resulting fractions were dried and resuspended in 20μl of 1% TFA.

Peptides were separated and analyzed by an UltiMate 3000 RSLC nano system coupled to an Orbitrap Fusion Tribrid mass spectrometer (both from Thermo Scientific). Peptides were firstly loaded onto an Acclaim PepMap300 trap column (300 µm x 5 mm) packed with C18 (5 µm, 300 Å) in loading buffer (0.1% trifluoroacetic acid in 2% acetonitrile) for 4 min at 15 μL/min and then separated in an EASY-Spray column (75 µm x 50 cm) packed with C18 (2 µm, 100 Å, Thermo Scientific) at a flow rate of 300 nL/min. Mobile phase A (0.1% formic acid in water) and mobile phase B (0.1% formic acid in acetonitrile) were used to establish a 60-min gradient from 4% to 35% B. Eluted peptides were ionized by electrospray.

Spectra were acquired by Orbitrap Fusion mass spectrometer (Thermo Scientific) with 3 seconds duty cycle. Full MS spectra were acquired in orbitrap within mass range 350- 1400 m/z with resolution 120 000 at 200 m/z and maximum injection time 50 ms. Most intense precursors were isolated by quadrupole with 1.6 m/z isolation window and fragmented using collision induced dissociation (CID) with collision energy set to 30%. Fragment ions were detected in ion trap with scan range mode set to normal and scan rate set to rapid with maximum injection time 50 ms. Fragmented precursors were excluded from fragmentation for 60 seconds. For quantificative information of TMT label, 10 most intense fragments were isolated (simultaneous precursor selection) and fragmented in higher-energy C-trap dissociation (HCD) on 65% energy, max accumulation time 140 ms, and fragments were measured in orbitrap on 60 K resolution (McAlister et al., 2014).

Raw data were processed in Max Quant 2.1. TMT reporter ions ratios were used for estimation of relative amount of each protein. The search was done against *Mus musculus* (Uniprot, 16981 entries), *L. mexicana* protein database (NCBI, 16299 entries), and common contaminant database. Modification was set: peptide N terminus, lysine (unimod nr:737) and cysteine (unimod nr:39) as static, and methionine oxidation (unimod: 1384), and protein N terminus acetylation (unimod: 1) as a variable. FDR threshold for peptide and protein identification was set to 1%. Proteins from mice and common contaminants were filtered off from the results. After filtering data were normalized to the median and transferred into binary logarithm. The differences between the individual samples (axenic – lesion-derived, axenic – macrophage-derived, macrophage-derived – lesion-derived) were investigated. Furthermore, only proteins whose content in the samples differed significantly by at least two-fold were selected. As a statistical test we used permutation-based false discovery rate analysis (FDR), FDR 0.05. Significance was considered as p < 0.05. Proteins without annotation or without known function were searched with BlastP on NCBI site https://blast.ncbi.nlm.nih.gov.

Proteins were divided into several functional groups (virulence factors, glycolysis, lipid metabolism, oxidative phosphorylation, amino acid metabolism, oxidative stress response, molecular motors, vesicular transport, cytoskeleton, transmembrane transport).

## Results

### Isobaric tag proteomic analysis

The proportions of *L. mexicana*, mouse and common contaminant proteins were determined in all samples by label free proteomic analysis. *Leishmania* peptides represent 70 to 90% and 30% of the total protein extract in axenic and macrophage-derived amastigotes, respectively. The percentage of peptides from lesion-derived amastigotes ranges from 9 to 14%, depending on the quality of the lesion and the accuracy of excision. The minimal content of *Leishmania* peptides in samples selected for our experiments was 10%.

**Figure 2:**
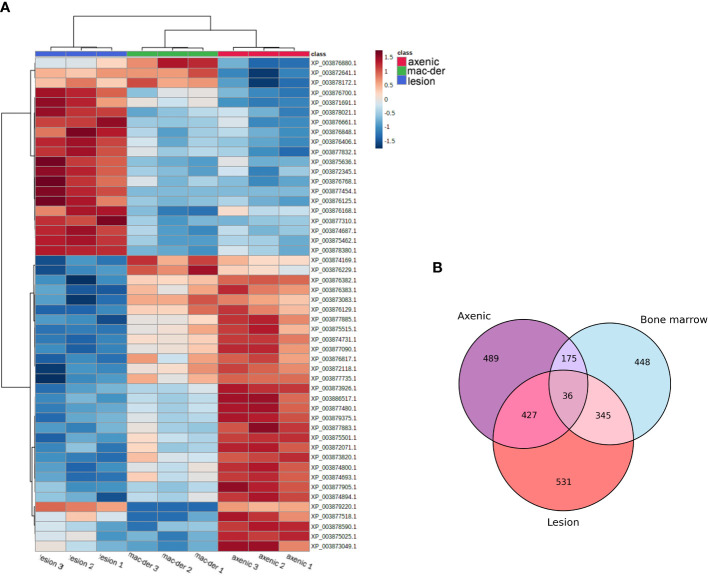
**(A)** Hierarchical Clustering Heat map representing the top 50 differentially expressed proteins in different amastigote type with biological triplicate. The protein abundance was normalized to enable the comparison of data obtained from LC-MS/MS. The MetaboAnalyst 5.0 website was used for data normalization and to generate the figure. The lines in the heatmap represent the relative abundance of metabolites across the samples of the three compared groups (lesion-derived, macrophage-derived, and axenic amastiogtes); each protein ID is indicated on the right side of the figure. The columns corresponding to the amastigote type are indicated at the top by color (blue for les-derived amastigotes, green for macrophage-derived amastigotes, and blue for axenic amastigotes). Each of the nine columns corresponds to one biological replicate (three per amastigote type). On the upper right side of the figure is a scale indicating the color code relative to the normalized protein abundance (ranging from -1.5 to 1.5). **(B)** Venn diagram indicates the overlap of differentially expressed proteins between amastigote types.

**Figure 3:**
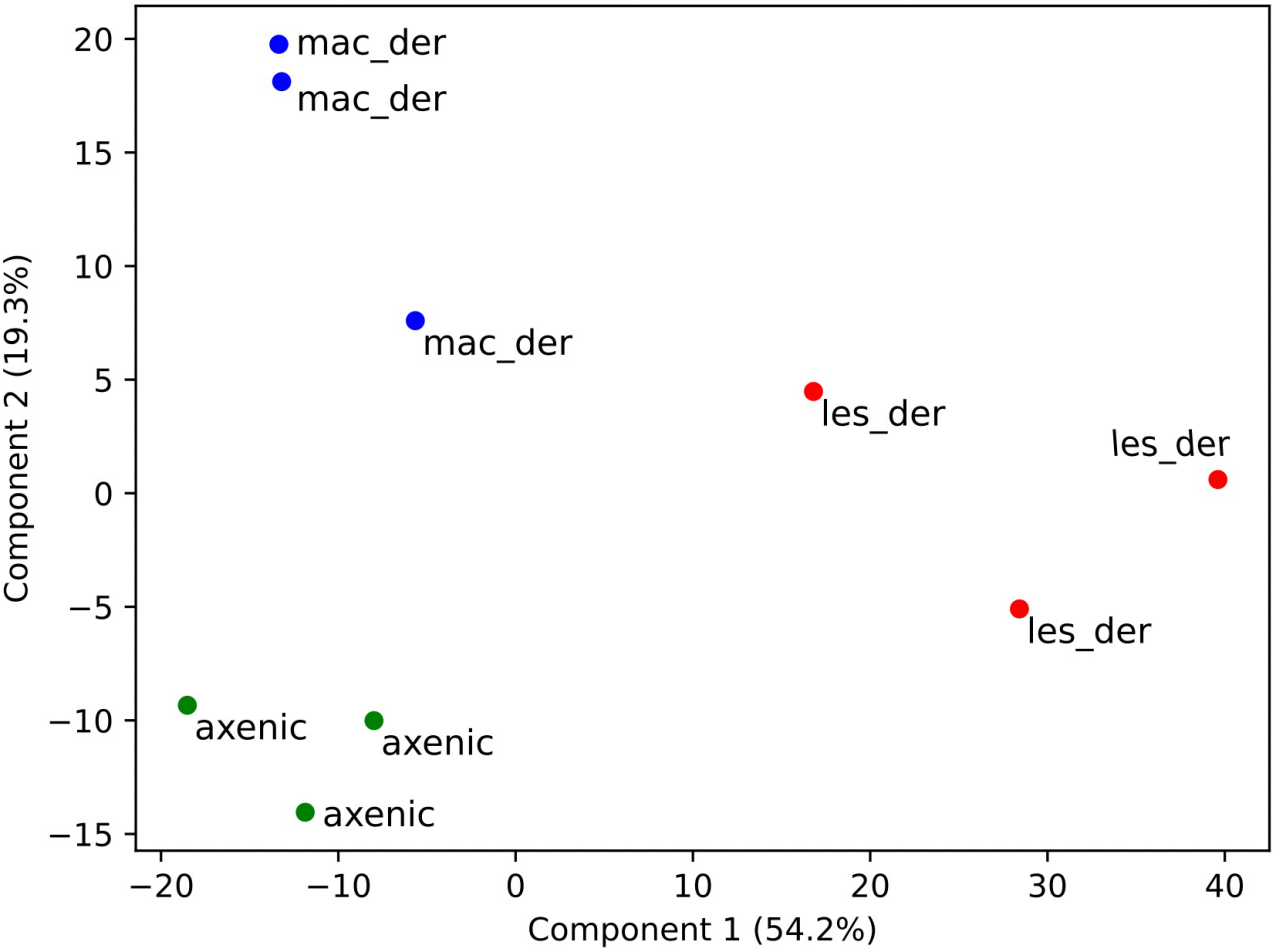
Clustering of samples after isobaric quantitative proteomics. Principal component analysis of axenic (green dots), macrophage-derived (blue dots) and lesion-derived (red dots) amastigotes prepared from 3 biological replicates.

A total of 3797 proteins were detected by proteomic analysis using isobaric tags. After filtering off common contaminants, mouse proteins and proteins assigned to both *Leishmania* and mouse identification, 1479 proteins of *L. mexicana* origin were obtained [*Leishmania mexicana* MHOM/GT/2001/U1103]. Hierarchical clustering of significantly altered proteins in at least one pairwise comparison was performed in ANOVA test and top 50 most altered proteins were represented as a heatmap using a platform MetaboAnalyst 5.0 (Pang et al., 2022). A heat map with clusterization shows that both alternative amastigotes are closer to each other than to lesion amastigotes (**Figure 2A**). Overlap between differentially expressed proteins between amastigote types is presented as Venn diagram (**Figure 2B**). The difference of various amastigote types was evaluated by Jaccard similarity index for each comparison (axenic-les-der = 72.01%, axenic-mac-der = 22.97%, mac-der-les-der = 54.42%. Reproducibility and uniformity of biological replicates preparation is demonstrated by principal component analysis (PCA) (**Figure 3**).

**Figure 4:**
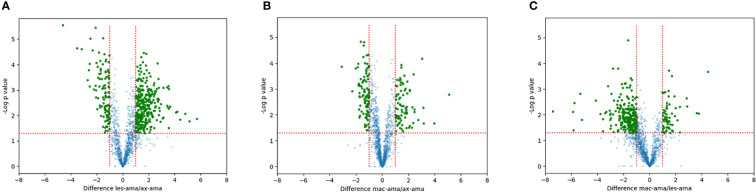
Quantitative proteomic analysis. Volcano plot visualization of differentially expressed proteins between **(A)** lesion-derived amastigotes vs. axenic. **(B)** macrophage-derived vs. axenic amastigotes. **(C)** macrophage-derived vs. lesion-derived amastigotes. Green dots correspond to proteins showing increased or reduced abundance (fold change ≥2, p-value < 0.05). Blue dots represent proteins that didn’t differ in each comparison.

Proteins showing fold changes ≥ 2 and p-value < 0.05 for each set of comparison were considered and displayed by volcano plots (**Figure 4**). Compared to axenic amastigotes 296 and 130 proteins were at least 2-fold more and less abundant in lesion-derived amastigotes (**Figure 4A**). In macrophage-derived amastigotes, 106 proteins were more abundant and 69 showed reduced abundance when compared to axenic amastigotes (**Figure 4B**). In lesion-derived amastigotes, 241 proteins were more abundant while 103 were decreasing compared to macrophage-derived amastigotes (**Figure 4C**).

### Basic functional groups of proteins

The 194 proteins quantified in our proteomic comparison were classified in functional groups (**Tables 1**–**10**) and revealed significant differences in protein abundance for several functions. Because the protein database [*Leishmania mexicana* MHOM/GT/2001/U1103] (Rogers et al., 2011) is relatively poorly annotated, several unassigned peptides had to be traced based on sequence similarity *via* https://blast.ncbi.nlm.nih.gov. We highlight those with known or expected function. The proteomic analysis revealed us some significant differences in protein expression among individual types of amastigotes.

**Table 1:** Virulence factors identified in all 3 comparisons that differ by at least 2-fold (T-test significant) between individual amastigote types.

Virulence factors				
Protein ID’s	Protein name	ax-les	ax-mo	mo-les
*XP_003872640.1*	putative amastin-like protein	-5,3	-2,5	-2,1
*XP_003874986.1*	glucose transporter, lmgt3	-6,1	-3,2	-1,9
*XP_003872328.1*	cysteine peptidase, Clan CA, family C19, putative	-4,6	-2,6	-1,8
*XP_003877237.1*	putative surface protein amastin	-3,3	-2,4	-1,4
*XP_003872630.1*	putative amastin-like protein B5	-13,4	-1,9	-7,2
*XP_003874822.1*	Prolyl oligopeptidase family protein [*L. donovani*]	-4,2	-1,5	-2,7
*XP_003872658.1*	putative cathepsin L-like protease	-4,1	1,1	-4,3
*XP_003886592.1*	amastin-like surface protein, putative [*L. donovani*]	-2,4	-1,1	-2,1
*XP_003873649.1*	cysteine peptidase, Clan CA, family C19,putative	-2,0	1,0	-2,1
*XP_003878921.1*	A600-3	-2,4	-1,7	-1,4
*XP_003872625.1*	Amastin surface glycoprotein, putative [*L. donovani*]	-1,4	-9,4	6,9
*XP_003878670.1*	elongation factor 1-beta	2,2	1,2	1,9
*XP_003876071.1*	cysteine peptidase, Clan CA, family C12,putative	2,2	1,7	1,3
*XP_003876623.1*	putative calpain-like cysteine peptidase, partial	2,8	1,4	2,0
*XP_003873458.1*	putative calpain-like cysteine peptidase	3,2	-1,2	4,0
*XP_003872882.1*	GP63, leishmanolysin	4,4	1,9	2,3

Color shade shows relative protein abundances among individual groups of amastigotes; red/green color represents at least two-fold up-/down-regulated proteins, yellow means non differing proteins.

**Table 2:** Glycolytic proteins identified in all 3 comparisons that differ by at least 2-fold (T-test significant) between individual amastigote types.

Glycolysis				
Protein ID's	Protein name	ax-les	ax-mo	mo-les
*XP_003873158.1*	glucose-6-phosphate isomerase	-2,5	1,1	-2,8
*XP_003872889.1*	glycerol-3-phosphate dehydrogenase [NAD+],glycosomal/mitochondrial	-2,2	-1,3	-1,7
*XP_003875668.1*	aldose 1-epimerase-like protein	1,4	2,4	-1,7
*XP_003875025.1*	2,3-bisphosphoglycerate-independent phosphoglycerate mutase	1,8	2,3	-1,3
*XP_003873490.1*	enolase	2,2	1,2	1,8
*XP_003875074.1*	phosphoglycerate kinase C, glycosomal	3,7	1,2	3,1

Color shade shows relative protein abundances among individual groups of amastigotes; red/green color represents at least two-fold up-/down-regulated proteins, yellow means non differing proteins.

**Table 3:** Proteins involved in lipid metabolism identified in all 3 comparisons that differ by at least 2-fold (T-test significant) between individual amastigote types.

Lipid metabolism				
*Protein ID’s*	Protein name	ax-les	ax-mo	mo-les
*XP_003877335.1*	succinyl-coa:3-ketoacid-coenzyme a transferase-like protein	-29,5	-12,9	-2,3
*XP_003876034.1*	Glycerophosphoryl diester phosphodiesterase family protein [*L. donovani*]	-7,2	-2,3	-3,1
*XP_003877379.1*	sphingosine phosphate lyase-like protein,putative	-3,7	-1,5	-2,5
*XP_003876021.1*	3-oxoacyl-(acyl-carrier protein) reductase,putative	-5,8	-3,8	-1,6
*XP_003874641.1*	putative ATP-binding cassette protein	-5,4	-3,9	-1,4
*XP_003872327.1*	enoyl-CoA hydratase/isomerase-like protein	-4,1	-4,4	1,1
*XP_003877753.1*	putative 3,2-trans-enoyl-CoA isomerase, mitochondrial precursor	-2,5	-4,4	1,8
*XP_003878080.1*	putative C-14 sterol reductase	-4,3	-1,8	-2,4
*XP_003875417.1*	choline dehydrogenase, like protein	-3,7	-1,2	-3,1
*XP_003876882.1*	putative propionyl-coa carboxylase beta chain	-3,4	1,0	-3,5
*XP_003877631.1*	putative monoglyceride lipase	-3,4	-1,3	-2,6
*XP_003874681.1*	Enoyl-CoA hydratase/isomerase family [*L. donovani*]	-3,3	1,5	-5,1
*XP_003879304.1*	putative glycerol kinase, glycosomal	-3,0	-1,2	-2,5
*XP_003873444.1*	putative fatty acid elongase	-2,7	1,1	-3,0
*XP_003872330.1*	Acyltransferase, putative [*L. donovani*]	-2,7	-1,3	-2,1
*XP_003877766.1*	putative lipase	-3,2	-1,7	-1,8
*XP_003877159.1*	alkyl dihydroxyacetonephosphate synthase	-3,0	-1,5	-2,0
*XP_003875399.1*	2-oxoisovalerate dehydrogenase alpha subunit, putative	-3,0	-1,8	-1,7
*XP_003871679.1*	glycerol-3-phosphate acyl transferase	-3,0	-1,8	-1,7
*XP_003877464.1*	3-hydroxy-3-methylglutaryl-CoA reductase,putative	-2,7	-1,5	-1,8
*XP_003874072.1*	putative peroxisomal enoyl-coa hydratase	-2,4	-1,4	-1,7
*XP_003877746.1*	putative 3,2-trans-enoyl-CoA isomerase, mitochondrial precursor	-1,9	-2,3	1,2
*XP_003873231.1*	Lipase_(class_3)_-_putative [*L. infantum*]	-1,5	-2,7	1,8
*XP_003876456.1*	trifunctional enzyme alpha subunit, mitochondrial precursor-like protein	-1,8	1,2	-2,3
*XP_003878332.1*	putative 2,4-dienoyl-coa reductase fadh1	-1,6	1,6	-2,6
*XP_003871572.1*	putative fatty acyl CoA syntetase 1	1,1	2,6	-2,3
*XP_003875783.1*	DHHC palmitoyltransferase family protein [*L. donovani*]	1,2	2,1	-1,7
*XP_003873926.1*	acyl-CoA-binding protein, putative [*L. panamensis*]	5,7	3,3	1,7

Color shade shows relative protein abundances among individual groups of amastigotes; red/green color represents at least two-fold up-/down-regulated proteins, yellow means non differing proteins.

**Table 4:** Proteins of oxidative phosphorylation identified in all 3 comparisons that differ by at least 2-fold (T-test significant) between individual amastigote types.

Oxidative phosphorylation				
*Protein ID’s*	Protein name	ax-les	ax-mo	mo-les
*XP_003875391.1*	putative ATP synthase	-6,3	-1,1	-5,5
*XP_003877677.1*	putative cytochrome c oxidase VIII (COX VIII)	-3,9	1,6	-6,3
*XP_003872264.1*	ATP synthase subunit C family protein [*L. donovani*]	-3,1	-1,1	-3,0
*XP_003876168.1*	putative cytochrome c oxidase VII	-2,9	1,6	-4,8
*XP_003876210.1*	putative ATPase of the ABC class family protein [*L. donovani*]	-2,8	-1,4	-2,0
*XP_003875445.1*	putative ATP synthase F1 subunit gamma protein	-2,2	-1,1	-2,0
*XP_003873529.1*	putative cytochrome-b5 reductase	-2,4	-1,4	-1,7
*XP_003872150.1*	putative cytochrome c1, heme protein, mitochondrial precursor	-1,7	2,0	-3,4
*XP_003879149.1*	putative reiske iron-sulfur protein precursor	-1,2	2,0	-2,3
*XP_003875062.1*	cytochrome c oxidase subunit I	-2,0	2,3	-4,6
*XP_003873171.1*	cytochrome c oxidase subunit IV	-1,6	2,1	-3,4
*XP_003876472.1*	putative cytochrome c oxidase subunit V	-1,3	2,2	-2,8
*XP_003875663.1*	putative cytochrome c oxidase subunit 10 [*L. braziliensis*]	-1,2	2,2	-2,8
*XP_003875439.1*	putative cytochrome c oxidase subunit VI	-1,2	2,7	-3,3
*XP_003872832.1*	putative cytochrome b5-like protein	1,5	3,1	-2,0
*XP_003877776.1*	ubiquinol-cytochrome-c reductase-like protein	2,2	2,3	-1,0
*XP_003874894.1*	ubiquinone biosynthesis methyltransferase,putative	4,1	2,1	2,0
*XP_003873820.1*	putative cytochrome c	48,3	5,2	9,2

Color shade shows relative protein abundances among individual groups of amastigotes; red/green color represents at least two-fold up-/down-regulated proteins, yellow means non differing proteins.

**Table 5:** Amino acid metabolism enzymes identified in all 3 comparisons that differ by at least 2-fold (T-test significant) between individual amastigote types.

Amino acid metabolism				
*Protein ID’s*	Protein name	ax-les	ax-mo	mo-les
*XP_003875729.1*	cytosolic leucyl aminopeptidase	-15,7	-2,4	-6,4
*XP_003877079.1*	serine hydroxymethyltransferase	-7,0	-2,5	-2,8
*XP_003873031.1*	3-methylcrotonoyl-CoA carboxylase beta subunit,putative	-5,2	-1,3	-4,0
*XP_003876604.1*	putative methylmalonyl-coenzyme a mutase	-4,2	-2,7	-1,6
*XP_003871691.1*	delta-1-pyrroline-5-carboxylate dehydrogenase, putative	-3,3	-2,2	-1,5
*XP_003871906.1*	dihydrolipoamide branched chain transacylase, putative	-3,1	-2,1	-1,5
*XP_003873627.1*	glutamate dehydrogenase	-2,7	-3,2	1,2
*XP_003878903.1*	amidinotransferase, putative [*L*. *panamensis*]	-2,4	-2,5	1,0
*XP_003877832.1*	methylcrotonoyl-coa carboxylase biotinylated subunitprotein-like protein	-4,7	-1,7	-2,8
*XP_003871917.1*	putative protein tyrosine phosphatase	-4,6	-1,7	-2,7
*XP_003873824.1*	Amidase, putative [*L. donovani*]	-4,4	-1,3	-3,5
*XP_003878177.1*	putative glucosamine-6-phosphate deaminase	-3,5	1,0	-3,6
*XP_003871973.1*	putative methylthioadenosine phosphorylase	-3,4	-1,5	-2,3
*XP_003875991.1*	Aminomethyltransferase folate-binding domain, putative [*L. donovani*]	-3,2	-1,5	-2,1
*XP_003872153.1*	cobalamin-dependent methionine synthase, putative	-2,8	-1,0	-2,7
*XP_003876680.1*	putative cysteine desulfurase	-2,7	1,1	-3,1
*XP_003877095.1*	putative acyl-CoA dehydrogenase	-2,5	-1,2	-2,1
*XP_003878111.1*	putative cystathionine beta-lyase	-3,5	-1,7	-2,0
*XP_003879269.1*	putative acyl-CoA dehydrogenase	-2,8	-1,9	-1,4
*XP_003879010.1*	putative 2-oxoisovalerate dehydrogenase beta subunit, mitochondrial precursor	-2,3	-1,8	-1,3
*XP_003872473.1*	putative guanine deaminase	-1,9	1,1	-2,1
*XP_003879319.1*	putative cystathione gamma lyase	1,4	-1,9	2,7
*XP_003877520.1*	putative 5-methyltetrahydropteroyltriglutamate–homocystein emethyltransferase	1,6	-9,7	15,8
*XP_003877630.1*	putative N-acyl-L-amino acid amidohydrolase	2,3	-3,9	8,7
*XP_003872049.1*	putative glutamine synthetase	3,6	1,9	1,9
*XP_003876383.1*	putative asparagine synthetase a	8,3	1,3	6,6
*XP_003877497.1*	S-adenosylmethionine synthetase	4,4	2,3	1,9

Color shade shows relative protein abundances among individual groups of amastigotes; red/green color represents at least two-fold up-/down-regulated proteins, yellow means non differing proteins.

**Table 6:** Oxidative stress response enzymes identified in all 3 comparisons that differ by at least 2-fold (T-test significant) between individual amastigote types.

Oxidative stress response				
*Protein ID’s*	Protein name	ax-les	ax-mo	mo-les
*XP_003874178.1*	putative gamma-glutamylcysteine synthetase	-23,1	-3,6	-6,5
*XP_003872375.1*	putative dihydrolipoamide dehydrogenase	-8,2	-1,7	-4,9
*XP_003872443.1*	tryparedoxin-like protein	-2,5	-1,1	-2,2
*XP_003872441.1*	tryparedoxin	2,1	1,7	1,3
*XP_003876381.1*	type II (glutathione peroxidase-like) tryparedoxin peroxidase	3,1	1,3	2,4
*XP_003876382.1*	type II (glutathione peroxidase-like) tryparedoxin peroxidase	3,8	1,5	2,6
*XP_003878590.1*	putative ascorbate-dependent peroxidase	6,0	6,2	-1,0

Color shade shows relative protein abundances among individual groups of amastigotes; red/green color represents at least two-fold up-/down-regulated proteins, yellow means non differing proteins.

**Table 7:** Molecular motors identified in all 3 comparisons that differ by at least 2-fold (T-test significant) between individual amastigote types.

Molecular motors				
*Protein ID’s*	Protein name	ax-les	ax-mo	mo-les
*XP_003879287.1*	dynein light chain, putative [*L. panamensis*]	-101,6	-4,8	-21,1
*XP_003876894.1*	putative dynein heavy chain	-79,2	-11,4	-7,0
*XP_003874247.1*	putative kinesin	-3,6	-1,0	-3,6
*XP_003874115.1*	putative dynein light chain 2B, cytoplasmic	-3,2	-1,8	-1,8
*XP_003878689.1*	putative myosin IB heavy chain	-2,1	-1,4	-1,5
*XP_003876152.1*	putative dynein heavy chain	1,0	-3,6	3,6
*XP_003873485.1*	putative kinesin K39	3,9	1,8	2,2
*XP_003886543.1*	putative kinesin [*L. major*]	3,9	2,1	1,8

Color shade shows relative protein abundances among individual groups of amastigotes; red/green color represents at least two-fold up-/down-regulated proteins, yellow means non differing proteins.

**Table 8:** Vesicular transport proteins identified in all 3 comparisons that differ by at least 2-fold (T-test significant) between individual amastigote types.

Vesicular transport				
*Protein ID’s*	Protein name	ax-les	ax-mo	mo-les
*XP_003879239.1*	putative phosphoinositide-binding protein	-3,2	-2,9	-1,1
*XP_003875197.1*	putative coatomer beta subunit	-2,7	-4,9	1,8
*XP_003878817.1*	putative adaptor complex subunit medium chain 3	-15,6	-1,9	-8,3
*XP_003878137.1*	putative SNAP protein [*L. major*]	-2,4	1,1	-2,7
*XP_003872565.1*	putative Qc-SNARE protein	-2,2	-1,5	-1,5
*XP_003876120.1*	putative epsin	2,7	1,2	2,3
*XP_003875036.1*	putative beta-adaptin	3,0	-1,2	3,6
*XP_003878067.1*	COP9 signalosome, subunit CSN8 family protein [*L. donovani*]	2,9	1,5	1,9

Color shade shows relative protein abundances among individual groups of amastigotes; red/green color represents at least two-fold up-/down-regulated proteins, yellow means non differing proteins.

**Table 9:** Cytoskeleton proteins identified in all 3 comparisons that differ by at least 2-fold (T-test significant) between individual amastigote types.

Cytoskeleton				
*Protein ID’s*	Protein name	ax-les	ax-mo	mo-les
*XP_003875956.1*	LIM domain family protein [*L. donovani*]	-30,0	-1,8	-16,7
*XP_003875419.1*	microtubule-binding protein, putative [*L. panamensis*]	-2,1	-2,3	1,1
*XP_003879352.1*	CRAL/TRIO domain family protein [*L*. *donovani*]	-1,1	-2,0	1,8
*XP_003878744.1*	p25-alpha, putative [*L. donovani*]	1,3	-2,8	3,5
*XP_003872679.1*	beta tubulin	2,6	-1,9	5,1
*XP_003878782.1*	putative G-actin binding protein	2,9	-1,6	4,7
*XP_003878811.1*	flagellar_attachment_zone_protein_putative|GeneDB : LmjF.34.2530 [*L. donovani*]	2,2	1,6	1,4

Color shade shows relative protein abundances among individual groups of amastigotes; red/green color represents at least two-fold up-/down-regulated proteins, yellow means non differing proteins.

**Table 10:** Transmembrane transport proteins identified in all 3 comparisons that differ by at least 2-fold (T-test significant) between individual amastigote types.

Transmembrane transport				
*Protein ID’s*	Protein name	ax-les	ax-mo	mo-les
*XP_003872500.1*	putative ABC transporter	-21,8	-6,5	-3,3
*XP_003878160.1*	putative ABC transporter	-4,9	-2,6	-1,8
*XP_003873076.1*	SEC61-like (pretranslocation process) protein,putative	-5,7	-1,5	-3,7
*XP_003874210.1*	intraflagellar transport protein component, putative	-4,6	-1,5	-3,0
*XP_003877640.1*	putative vacuolar-type proton translocating pyrophosphatase 1	-4,2	-1,7	-2,5
*XP_003875636.1*	putative vacuolar type h+ ATPase subunit	-3,1	1,1	-3,3
*XP_003871665.1*	putative mitochondrial carrier protein	-2,6	1,1	-2,8
*XP_003872210.1*	putative vacuolar-type Ca2+-ATPase	-2,4	1,1	-2,5
*XP_003872932.1*	Archaic_translocase_of_outer_membrane_12_kDa_subunit [*L. braziliensis*]	-2,1	1,1	-2,3
*XP_003879439.1*	putative mitochondrial phosphate transporter	-2,9	-1,5	-1,9
*XP_003876106.1*	putative ABC transporter	-2,3	-1,4	-1,7
*XP_003871558.1*	putative poly(A) export protein	-2,0	-1,5	-1,3
*XP_003875664.1*	ABC transporter-like protein	-1,3	-2,2	1,7
*XP_003874875.1*	vacuolar protein sorting-associated protein-like protein	1,8	-1,1	2,0
*XP_003871644.1*	voltage-dependent anion-selective channel, putative [*L. panamensis*]	1,9	2,1	-1,1
*XP_003874566.1*	ER–golgi transport protein erv25 precursor, putative	2,2	1,3	1,7

Color shade shows relative protein abundances among individual groups of amastigotes; red/green color represents at least two-fold up-/down-regulated proteins, yellow means non differing proteins.

### Virulence factors

Higher expression of amastins, glucose transporter 3 (GT3), cysteine proteinases (CP) and propyl oligopeptidase family proteins was observed in lesion-derived amastigotes compared to axenic and macrophage-derived ones. Conversely, the highest expression of gp63 was shown in axenic amastigotes and lowest in lesion-derived amastigotes. Protein A600-3, showed higher expression in lesion-derived amastigotes than in axenic amastigotes (**Table 1**).

### Glycolysis

Generally, the most pronounced expression of glycolytic enzymes was observed in axenic amastigotes and amastigotes isolated from macrophages (e.g., enolase, phosphoglycerate kinase). An exception was found among some glycolytic enzymes located in glycosomes that were more expressed in amastigotes from the lesion compared to axenic and macrophage-derived ones (e.g., glucose-6-phosphate isomerase, glycerol-3-phosphate dehydrogenase). Expression of 2,3-phosphoglycerate mutase was significantly higher in axenic amastigotes compared to macrophage-derived amastigotes. Aldose 1-epimerase was upregulated in macrophage-derived amastigotes compared to lesion-derived and axenic amastigotes (**Table 2**).

### Lipid metabolism

Expression of enzymes involved in lipid metabolism was mostly higher in lesion-derived amastigotes compared to both alternative types of amastigotes. An example is in basic beta-oxidation enzymes (e.g. enoyl-CoA hydratase/isomerase, 3,2-trans-enoyl-CoA isomerase, acyl-CoA dehydrogenase) as well as in other enzymes involved in β-oxidation indirectly (lipase, acyl CoA synthetase, acyltransferase, C-14 sterol reductase, propionyl-CoA carboxylase and long-chain-fatty-acid-CoA ligase). Furthermore, some anabolic enzymes (e.g. glycerol kinase) were upregulated in lesion-derived amastigotes compared to axenic and macrophage-derived ones (**Table 3**).

### Oxidative phosphorylation

Enzymes of respiratory chain were generally more expressed in lesion-derived amastigotes compared to other types of amastigotes. This applies for example to ATP synthase, succinate dehydrogenase, cytochrome c reductase. Interestingly most peptides identified as cytochrome c oxidase subunits showed downregulation in macrophage-derived amastigotes in comparison to axenic amastigotes and lesion-derived ones. Axenic amastigotes expressed the highest amount of the electron carrier protein cytochrome c and ubiquinone biosynthesis methyltransferase (**Table 4**).

### Amino acid metabolism

The most of detected enzymes of amino acid catabolism was upregulated in lesion-derived amastigotes in comparison to axenic and macrophage-derived ones. In general, expression of catabolic enzymes was lowest in axenic amastigotes. Detected enzymes involved in amino acid catabolism are e. g. serine hydroxymethyl transferase, methylmalonyl CoA mutase, dihydrolipoamide branched chain transacylase, 2-oxoisovalerate dehydrogenase, delta-1-pyrroline-5-carboxylate dehydrogenase, glutamate dehydrogenase, methylthioadenosin phosphorylase, lysine decarboxylase. The most upregulated anabolic enzyme of lesion-derived amastigotes was methionine synthase. In macrophage-derived amastigotes, anabolic cystathione gamma lyase and 5-methyltetrahydropteroyltriglutamate-homocysteine methyltransferase involved in methionin metabolism and catabolic N-acyl-L-amino acid amidohydrolase were upregulated. In axenic amastigotes, mostly anabolic enzymes such as glutamine synthetase, asparagine synthetase and S-adenylmethionine synthetase were upregulated (**Table 5**).

### Oxidative stress response

Gamma-glutamylcysteine synthetase was upregulated in lesion-derived amastigotes and least expressed in axenic amastigotes. The same pattern of expression shows dihydrolipoamide dehydrogenase. Tryparedoxin, thioredoxin, type II (glutathione peroxidase-like) tryparedoxin peroxidase and ascorbate-dependent peroxidase manifested upregulation in axenic amastigotes. Macrophage-derived amastigotes possessed very similar expression pattern as axenic amastigotes (**Table 6**).

### Molecular motors, vesicular transport and cytoskeleton

Molecular motors were highly expressed in lesion-derived amastigotes. Dynein and kinesin were both upregulated in lesion-derived amastigotes, while dynein was least expressed in axenic amastigotes. Kinesin was least expressed in macrophage-derived amastigotes (**Table 7**). Generally, the highest expression of proteins associated with vesicle transport was observed in lesion-derived amastigotes (**Table 8**). A similar trend was seen in cytoskeleton-associated proteins (**Table 9**).

### Transmembrane transport

Expression of most proteins associated with transmembrane transport was upregulated in lesion-derived amastigotes. The most abundant proteins found upregulated in lesion-derived amastigotes were ATP binding cassette (ABC) transporters (**Table 10**).

**Figure 5:**
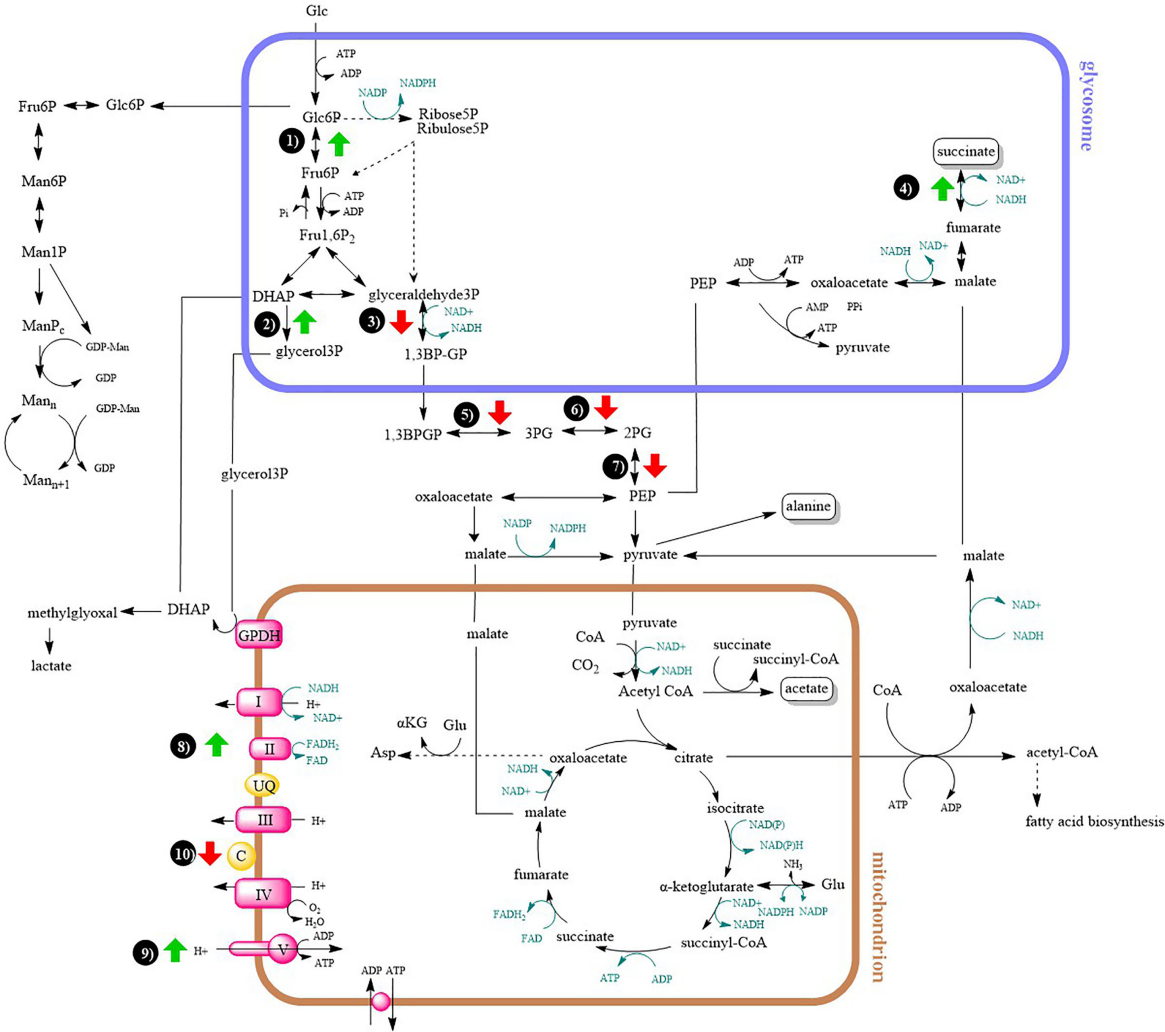
Metabolic map of central carbon metabolism and oxidative phosphorylation. Arrows indicates at least two-fold up- or downregulation (T-test significant) of enzyme in lesion-derived amastigotes compared to axenic amastigotes (macrophage-derived amastigotes are usually intermediate between the two). 1) glucose-6-phosphate isomerase 2) glycerol-3-phosphate dehydrogenase 3) glyceraldehyde-3-phosphate dehydrogenase 4) fumarate reductase 5) phosphoglycerate kinase 6) phosphoglycerate mutase 7) enolase 8) succinate dehydrogenase 9) ATP synthase 10) cytochrome c.

For a better clarity and comprehensive insight, differences in energy metabolism have been shown in a metabolic map (**Figure 5**).

## Discussion

Previous comparative studies on *Leishmania* stages were mostly based on genomic and transcriptomic methods (Peacock et al., 2007; Fiebig et al., 2015; De Pablos et al., 2019). Nevertheless, proteomic studies in kinetoplastids are essential because regulation of gene expression occurs mainly post-transcriptionally (Karamysheva et al., 2020; Piel et al., 2022). First proteomic studies were focused especially on comparison of promastigote and amastigote stages and were based on 2 DE-gel and subsequent mass spectrometer analysis (Walker et al., 2006; Paape et al, 2008). However, this method allows only comparison of proteins with high abundancy while membrane proteins are highly underrepresented. Advances in proteomic methods allowed comparison of relative protein abundances and isobaric labeling used in this manuscript started to be used for protein quantification. A big advantage of this „shotgun” proteomic method is that it allows parallel detection of thousands of peptides in a single mass spectrometry run. Quantification *via* tandem mass tags benefits from sample multiplexing, it allows to detect fewer missing values compared to label free methods as well as detection of peptides with low abundancy in a cell (Thompson et al, 2003). This method has been previously used in order to compare differential expression of proteins in membranes of promastigote and amastigote or changes in protein expression in different time points of promastigote to amastigote transformation (Rosenzweig et al., 2008; Lynn et al., 2013). We used this proteomic approach to evaluate differences in proteome of lesion-derived amastigotes in comparison to axenic and macrophage-derived ones widely used in experimental assays.

Although the level of protein doesn’t systematically reflect the mRNA abundance, study made by Fiebig et al. (2015) presented transcriptomic data suggesting closer resemblance of axenic amastigotes to promastigotes rather than to macrophage-derived amastigotes. Another transcriptomic study showed that lesion-derived amastigotes significantly differ in expression of proteins compared to promastigotes and, importantly, axenic amastigotes were again closer to promastigotes (Holzer et al., 2006).

In our study, lesion-derived amastigotes showed upregulation of some important virulence factors and metabolic pathways typical for amastigote stage, particularly amastins, GT3 and A600-3 protein and CP´s. The highest GT3 expression in amastigotes from an infected animal can be considered as an adaptation to lower sugar levels in the environment, to cover necessary sugar intake for the amastigote stage. This was supported by Feng et al. (2011), suggesting that the expression of GT3 and other similar proteins is regulated by the availability of glucose in the environment. A600-3 protein, a potential virulence factor (Murray et al., 2010), showed significantly higher expression in these amastigotes compared to axenic amastigotes. Higher expression of cysteine ​​proteinases was also observed in amastigotes from the lesion. This is consistent with the fact that most CPs had higher expression in amastigotes compared to promastigotes (Mottram et al., 1992; Souza et al., 1992); increased expression is probably required for modulation of the host cellular response (Cameron et al., 2004). CPB inhibits Th1 protective immune response in mice (Buxbaum et al., 2003) and its function is also associated with autophagy, a process that is required for transformation to amastigote form (Denise et al., 2003; Williams et al., 2006). A higher CPB expression in lesion-derived amastigotes could be also related to larger megasome volumes in amastigotes from the infected animal (Ueda-Nakamura et al., 2007). Prolyl oligopeptidase family protein is another potential virulence factor upregulated in lesion-derived amastigotes compared to axenic and macrophage-derived ones, it seems to be involved in macrophage invasion process as found in *L. infantum* (Lasse et al., 2020).

As a carbon source, amastigotes prefer sugars, if available (McConville et al., 2015). Due to lower availability of sugars in parasitophorous vacuole, their supply to the amastigote cell is significantly reduced. In our comparative study, lower expression of glycolytic enzymes was generally observed in lesion-derived amastigotes compared to axenic and macrophage-derived ones, as expected by the higher availability of sugars in media supplemented with bovine serum compared to host environments where sugar intake may fluctuate. As an exception, some glycolytic enzymes located in glycosomes were more expressed in amastigotes from the lesion (e. g. glucose-6-phosphate isomerase). This is consistent with a study of Rosenzweig et al. (2008), where expression of glycolytic enzymes located in the glycosome of *L. donovani* was increased during transformation into amastigotes, while the expression of cytosolic glycolytic enzymes was decreased (e. g. enolase, phosphoglycerate mutase). Enzymes co-located in glycosomes may be important in the rapid response to a change in carbon source availability (Rosenzweig et al., 2008).

Since glyoxylate cycle is absent in *Leishmania* parasites, they are unable to switch to the utilization of fatty acids as a sole carbon source (Saunders et al., 2014; McConville et al., 2015). Amastigotes, contrary to promastigotes, overexpress enzymes involved in metabolism of fatty acids, which are the second preferred source of carbon (Hart and Coombs, 1982; Paape et al., 2010; Saunders et al., 2011; Saunders et al., 2014). Results of this study indicate the most pronounced expression of β-oxidation enzymes in amastigotes from the host lesion in comparison with the remaining types of amastigotes, which is in accordance with Rosenzweig et al., (Rosenzweig et al., 2008). The expression of other enzymes involved in β-oxidation indirectly (lipase, acyl CoA synthetase) was also increased in lesion-derived amastigotes compared to alternatively cultured amastigotes. Expression of glycerol kinase was increased in lesion amastigotes, suggesting that glycerol produced by lipases is further processed in gluconeogenesis (Opperdoes and Coombs, 2007; Rodriguez-Contreras and Hamilton, 2014), which is essential in amastigote stage living in sugar depleted conditions (Naderer et al., 2006).

Amastigotes proliferate in amino acid rich environment (Naderer and McConville, 2008) and therefore catabolism of some amino acids is increased compared to promastigote stages (Rosenzweig et al., 2008). This fact was reflected in lesion-derived amastigotes, where expression of most enzymes connected with amino acid catabolism was increased. An example is glutamate dehydrogenase, where increased expression has been measured in *L. donovani* amastigotes (Rosenzweig et al., 2008).

Furthermore, the fact that enzymes of oxidative phosphorylation and enzymes of energetic metabolism were more highly expressed in lesion-derived amastigotes compared to other types of amastigotes is in accordance with previously done studies (Rosenzweig et al., 2008; Saunders et al., 2014). The pathways of oxidative phosphorylation and β-oxidation are essential for the virulence of amastigotes, and when they are disrupted, virulence is lost (Dey et al., 2010; Gannavaram et al., 2012).

Overall higher expression of proteins involved in signalization, vesicular trafficking and membranous transport was observed in lesion-derived amastigotes in comparison to alternative types. Vesicular transport has been shown to be very important for survival in the intracellular environment due to the extraction of nutrients from the extracellular environment, the release of virulence factors, metabolites and drug resistance molecules in pathogens generally as well as hijacking the immune response (Silverman et al., 2010; Atayde et al., 2015; Bouvy et al., 2017; Coakley et al., 2017). In a study comparing protein expression of promastigote vs. amastigote, an increased expression of dynein was observed in amastigote form (Biyani and Madhubala, 2012). Also in our study, most peptides identified as dynein were more expressed in lesion-derived amastigotes.

In contrast to lesion-derived amastigotes, axenically cultivated amastigotes had downregulated some virulence factors specific for amastigote stage. Good examples are amastins with proven function in mediation of contact with the membrane of parasitophorous vacuole which is absent in axenic amastigotes (de Paiva et al., 2015). Lower expression of A600 protein family in axenic amastigotes may indicate their closer resemblance to promastigotes as A600 protein family is typically expressed in amastigote stage (Bellatin et al., 2002). In axenic amastigotes, there is no interaction with the host, and in amastigotes isolated from macrophages *ex vivo*, the environment is depleted of interaction with other immune cell types which could be the reason for the lowest expression of CPB. Other cysteine ​​proteinases (e. g. the CA clan) were also identified in our samples, whose expression was higher alternately in all types of amastigotes, but whose function in *Leishmania* parasites is not yet well known (Siqueira-Neto et al., 2018) and therefore cannot be further evaluated with respect to these results.

CPB regulates the expression of another virulence factor – gp63 (Casgrain et al., 2016). Gp63 is present in both amastigotes and promastigotes. It is known that the expression of surface form of gp63 is higher in promastigotes compared to amastigotes of *L. mexicana* (Medina-Acosta et al., 1989). Along with a function as a virulence factor gp63 plays a role in evasion of complement-mediated lysis, induction of phagocytosis by host macrophage, degradation of extracellular matrix, inhibition of natural killer cellular functions, degradation of macrophage cytosolic proteins and it helps intracellular amastigotes to survive within parasitophorous vacuole (Russell and Wilhelm, 1986; Chaudhuri and Chang, 1988; Chaudhuri et al., 1989; Brittingham et al., 1995; Seay et al., 1996; Brittingham et al., 1999; McGwire et al., 2003; Kulkarni et al., 2006; Hallé et al., 2009; Contreras et al, 2010). In our study, the highest expression of gp63 was recorded in axenic amastigotes. However, part of our experiments did not determine the location of the identified proteins, so it is not possible to say with certainty whether the overall higher expression of gp63 is an indicator of the similarity of axenic amastigotes with promastigotes. Elongation factor alpha found in exosomes possesses immunosuppressive properties and also plays a role in activation of host cells for invasion (Nandan et al., 2002). We observed highest expression of elongation factor alpha in macrophage-derived amastigotes. This protein has a role in translation, apoptosis and regulation of ubiquitine-mediated lysis (Gonen et al., 1996). It has been shown to interact with host SHP-1 which leads to downregulation of inducible nitric oxide synthase in activated macrophage (Nandan et al., 2002). Compared to amastigotes from the lesion, macrophage-derived amastigotes may have more active translation and generally higher expression of translation factors, that may explain their faster multiplication.

Some metabolic pathways are considered therapeutic targets for potential antileishmanial drugs (Sundar and Singh, 2018). Differential expression of proteins in alternative amastigotes´metabolism may cause various reactions to the drug when compared to natural conditions. The functional mechanism of some drugs is based on the modulation of macrophage function, supporting the need of intracellular amastigotes (Da-Silva et al., 1999). Also, higher expression of ABC proteins in amastigotes found in hosts can cause a different reaction to some drug compounds (e.g. by a higher degree of resistance) compared to alternative forms (Leprohon et al., 2006).

In conclusion, this study suggests that alternative methods of obtaining amastigotes do not result in a complete conversion equal to amastigotes developed in the host lesion. Expression patterns of macrophage-derived amastigotes were mostly between lesion-derived amastigotes and axenic amastigotes which suggests that they may be closer to natural model of infection and therefore more relevant model for experimental studies than axenic amastigotes.

## Data availability statement

The data presented in the study are deposited in the PRIDE repository (Perez-Riverol et al., 2019), accession number PXD036664.

## Ethics statement

The animal study was reviewed and approved by The Committee on Ethics of Laboratory Experiments, Faculty of Science, Charles University, Czech Republic.

## Author contributions

TL, LP, and KH contributed to conception and design of the study. PV acquired funding. LP, TL, and KH performed the experiments. KH performed the statistical analysis. LP wrote the first draft of the manuscript and prepared all tables. LP and KH prepared the figures. All authors reviewed and edited the manuscript. All authors contributed to the article and approved the submitted version.

## Funding

LP, PV, and TL are supported by the Ministry of Education of the Czech Republic through the ERD Fund, project CePaViP (project CZ.02.1.01/0.0/0.0/16_019/0000759) and by Research center UNCE (project no. 204072). The funders had no role in the study design, data collection and analysis, decision to publish, or preparation of the manuscript.

## Acknowledgments

We are grateful to Pavel Talacko from the Laboratory of Mass Spectrometry, Biocev, Charles University, for the assistance with sample preparation for mass spectrometry analyses. We would like to thank Vit Dvorak for language revision. We would also like to thank to Pascal Pescher who helped to improve the quality of presented work.

## Conflict of interest

The authors declare that the research was conducted in the absence of any commercial or financial relationships that could be construed as a potential conflict of interest.

## Publisher’s note

All claims expressed in this article are solely those of the authors and do not necessarily represent those of their affiliated organizations, or those of the publisher, the editors and the reviewers. Any product that may be evaluated in this article, or claim that may be made by its manufacturer, is not guaranteed or endorsed by the publisher.
